# Current bioinformatic approaches to identify DNase I hypersensitive sites and genomic footprints from DNase-seq data

**DOI:** 10.3389/fgene.2012.00230

**Published:** 2012-10-31

**Authors:** Pedro Madrigal, Paweł Krajewski

**Affiliations:** Laboratory of Biometry, Institute of Plant Genetics, Polish Academy of SciencesPoznań, Poland

## Chromatin accessibility

The formation of regions of open chromatin or nucleosome loss in eukaryotic genomes is an important factor elucidating potential regulatory activity. Nucleosome packaging, which organizes the DNA structure, acts as a regulator of transcription by enabling or restricting protein binding, and therefore facilitating the replication and coordination of gene activity (Cockerill, 2011). In addition, chromatin accessibility, which has been determined traditionally by regions of “open” or “closed” conformation, is subjected to dynamically changing events at accessible *cis*-regulatory elements (Bell et al., 2011).

Chromatin accessibility can be examined by DNase I digestion, and then uncovered by the DNase I cleavage pattern (Wu et al., 1979). The combination of DNase I digestion and high-throughput sequencing (DNase-seq) has been used to map chromatin accessibility *in vivo* in a given tissue or cell-type on a genome-wide scale (Song and Crawford, 2010). This technique allows for an unprecedented increase both in resolution and the range spanned, compared to the pre-next generation sequencing era (Kodama et al., 2007). The current DNase-seq protocol has been adapted from the methodology described by Boyle et al. (2008a), achieving higher resolution than DNase-chip, and can be applied to any species with a sequenced genome.

Although, the analysis of data coming from sequencing technologies such as chromatin immunoprecipitation followed by sequencing (ChIP-seq), or whole transcriptome shotgun sequencing (RNA-seq) have concentrated a huge level of research effort, methodologies for the analysis of DNase-seq data are relatively immature (Song and Crawford, 2010). This data presents its own peculiarities and should not be merely treated as ChIP-seq data, but instead linked to it to provide biological insights of chromatin domains and transcriptional regulation. The general view conceives regions of open chromatin spanning nucleosome-free or nucleosome-depleted regions often in the vicinity of transcription factor binding events.

## DNase I hypersensitive sites

DNase I hypersensitive sites (DHSs) indicate regions of an open chromatin state obtained as DNase-seq highly reproducible tag-enriched sites. The coverage formed by reads mapped uniquely, after artifact filtering (Baek et al., 2012), can be obtained as a standard format file, and visualized in a genome browser. The obtained profiles resemble to some extent the ones usually obtained by ChIP-seq, but there are several important differences: (1) Whereas ChIP data relate to a two-state situation of “bound” or “unbound” regions, DNase I acts as a generic indicator of chromatin state, and allows the handling of multiple states of chromatin accessibility (Shu et al., 2011); (2) ChIP-seq analyzers can employ two strand-specific approaches for peak detection: tag shifting or tag extension. Both strategies will hide the actual location of protein-DNA binding within a DHS; and (3) ChIP-seq peaks for a transcription factor are usually well-defined and can be identified by visual inspection, whereas DHSs are less evident due to tag enrichment over wide stretches of genomic sequence.

It is important to stress that there are two influencing factors that can change the DNase pattern: (1) How accessible is the region, determined by the fold-enrichment of the DHS and (2) How protected is the sequence where a transcription factor is binding (depth of the footprint). Therefore, the utilization of a ChIP-seq peak finder does not completely fit the patterns formed in a DNase-seq assay. However, due to the lack of well-established algorithms to handle DNase-seq data, popular ChIP-seq peak finders are used instead to pinpoint DHSs (Zhang et al., 2008; He et al., 2012). Among those peak callers, only F-seq (Boyle et al., 2008b) considers an algorithm adjustment specially dedicated to identify DHSs in its kernel density estimation approach, concerning the average fragment size of the experiment. The DHSs reported by this program have helped to integrate and interrelate data among several platforms (Shu et al., 2011; Song et al., 2011), for instance aiding the correlation *in vivo* of footprints with ChIP-seq enrichment (Boyle et al., 2011). F-seq has been also used to identify enriched sites in formaldehyde-assisted isolation of regulatory elements followed by sequencing (FAIRE-seq), but without any available statistical assessment concerning false discovery rate (FDR) or *p*-value calculation, with the DHSs obtained under different qualitative cutoffs, depending on a user-defined standard deviation threshold over the average signal with respect to a local background (Gaulton et al., 2010). As a consequence, the F-seq users need to employ time and effort on designing a proper statistical test for their experiment (Zhang et al., 2012).

Opposed to the F-seq approach, in Baek et al. (2012), the read extension to the average fragment size of the experiment is recommended. In this method, mappability-adjusted z-scores enable one to obtain statistical significance for the list of DHSs reported, to our knowledge being the first DNase-seq algorithm reporting FDR values for a list of DHSs. However, these two methodologies do not allow the inclusion of control samples in the analysis which, as has been demonstrated for ChIP-seq, can potentially reduce the FDR. Therefore, new statistical algorithms should be developed to exploit the potential of DNase-seq data more efficiently than, as is the current approach, peak callers developed originally for the analysis of ChIP-seq datasets (some of such tools are reviewed in Wilbanks and Facciotti, 2010).

## Footprint detection

At a very high sequencing depth it is possible to identify depleted narrow regions in the DHSs core, corresponding to protein footprints, ranging typically from 8 to 30 bp. Both the kernel density estimation approach (Boyle et al., 2008b) and the hotspot detection algorithm (Baek et al., 2012) will smooth the tag density profile and report the location of DHS peaks, making difficult the visualization and detection of confined depleted regions protected against DNase I cleavage. This problem can be solved by using DNase I cuts (read-start sites) for coverage determination instead of full-size or extended aligned reads. Thus, additional software to identify protein-DNA footprints is much needed. With this in mind, Hesselberth et al. (2009), presented a computational algorithm able to detect substantial DNase I cleavage reduction in the tag density compared to its adjacent flanking regions at nucleotide resolution. After computing depletion scores, non-overlapping footprints within intergenic regions have been reported. However, this method does not scale well for large genomes (Baek et al., 2012). Its modified version was introduced by Chen et al. (2010) who used a generalization of hidden Markov models and Bayesian networks, and considered non-uniquely mappable regions as missing data. This improved the precision of their previous approach in terms of FDR.

With sufficiently deep sequencing, the so-called “digital genomic footprinting” technique can reveal single protein-binding events (Hesselberth et al., 2009). Unlike ChIP-seq, which is specific for the protein under study, footprints identify narrow DNA regions that can be bound by any factor (Hager, 2009), showing significant enrichment for known motifs upstream of the transcription start sites (TSSs).

## Differential DNase I hypersensitivity

With the popularization and drastic cost decrease of sequencing leading to the generation of multiple sequenced samples, quantitative analysis of differential ChIP-seq binding across conditions, time stages or different tissues has been the subject of a great amount of research in the last 2 years (Bardet et al., 2011; Liang and Keles, 2012). However, adapting DNase-seq data singularities for differential analysis has just begun to be approached (He et al., 2012). The only proposed methodology computes scores of stimulus-dependent DHS changes, proving the utility of quantitative measures of chromatin accessibility differences between conditions to predict transcription factor binding. Coupling information of known motifs found within the DHS can improve the prediction, and using instead the changes in DHS (ΔDHS), produces the best prediction. These results are coherent with the theory that the interaction between a specific sequence and a transcription factor may be guided by different types of chromatin configuration (van Steensel, 2011).

## Data integration

Apart from the usual structural annotation and downstream analysis (including enrichment of known motifs or *de novo* motif discovery, with the canonical motif placed typically in the peak of a DHS) of the regions of interest, both for footprints or DHSs, the combination with other genomic data sources can unravel a plethora of novel biological insights. DHSs have positive correlation with active histone marks, whereas the correlation is negative for repressive histone marks, and DHSs score is higher for active genes than for silent ones (Shu et al., 2011). Furthermore, it has been shown recently that DNase-seq data, aided by regulatory genome sequences, can predict gene expression in a cell-type specific fashion (Natarajan et al., 2012). The utilization of prior knowledge can group the footprints or DHSs into more biologically meaningful target clusters, allowing a better understanding of how chromatin accessibility affects TF-DNA interaction. Although, the spatial distribution of DHSs/footprints is highly informative about binding, no one data source is fully enlightening when taken alone. For example, Centipede (Pique-Regi et al., 2011) improves TF-binding prediction by scanning the genome in search for known motifs or positional weight matrices, and integrating evolutionary sequence conservation, proximity to the nearest TSS, DNase I cuts, and histone modifications data into a Bayesian mixture model. However, not all factors influence the model in the same way: histone marks do not significantly improve the predictive power of DNase I accessibility. Centipede also shows the potential to extract quantitative measures of TF-binding from DNase-seq data. The disadvantage of Centipede is the compulsory requirement to know *a priori* the consensus sequence (motif) for each TF, which makes DNase-seq, if we consider the current state-of-the-art, a complementary tool of ChIP-seq rather than an independent assay to determine TF-binding sites genome-wide.

The correlation between gene expression and active and repressive histone marks have revealed four distinct modes of chromatin structure in humans, further invalidating the simplistic assumption that chromatin can only be in an “open” or “closed” conformation (Shu et al., 2011). Additionally, a cross-validated set of DNase-seq and FAIRE-seq sites allowed the creation of high-confidence open chromatin maps (Song et al., 2011). From this year, a manually curated web-server storing DNase-seq and ChIP-Seq data from human and mouse studies is publicly available (Qin et al., 2012).

New open questions should redirect the efforts to adapt each methodology to fruitfully map chromatin accessibility by DNase-seq, from the former stages of getting significant broad DNase I hypersensitive regions or narrow footprints, to the latter steps that include the differential assessment of chromatin accessibility changes and the correlation with other available genomic data. The question whether DNase-seq will eventually serve as a substitute for ChIP-seq, and to what extent, will be unraveled in the upcoming years.
